# Prognostic Factors in the Treatment of Advanced Endometrial Cancer Patients: 12-Year Experience of an ESGO Certified Center

**DOI:** 10.3390/cancers18020343

**Published:** 2026-01-22

**Authors:** Dimitrios Zouzoulas, Iliana Sofianou, Efthalia Markopoulou, Tilemachos Karalis, Kimon Chatzistamatiou, Vasilis Theodoulidis, Maria Topalidou, Eleni Timotheadou, Grigoris Grimbizis, Dimitrios Tsolakidis

**Affiliations:** 1 1st Department of Obstetrics & Gynecology, Aristotle University of Thessaloniki, “Papageorgiou” Hospital, 56429 Thessaloniki, Greece; 2 Radiotherapy Department, “Papageorgiou” Hospital, 56429 Thessaloniki, Greece; 3 Department of Oncology, Aristotle University of Thessaloniki, “Papageorgiou” Hospital, 56429 Thessaloniki, Greece

**Keywords:** advanced endometrial cancer, histological subtype, lymphovascular space invasion

## Abstract

(1) Background: Advance-stage endometrial cancer is a rare disease that encompasses a heterogeneous group of patients. Primary surgery is the treatment of choice, while neoadjuvant chemotherapy (NACT) seems to be an alternative option for inoperable frail patients. The aim of this study was to identify possible prognostic factors for advance-stage endometrial cancer patients. (2) Methods: We retrospectively analyzed the records of patients with endometrial cancer that underwent surgery in the 1st Department of Obstetrics–Gynecology from 2012 to 2023. Patients with advance-stage disease (FIGO stage III–IV) were included, while those with incidental microscopic lymph node metastases after staging lymphadenectomy were excluded. (3) Results: The population of this study consisted of 89 women. Patients were obese, with moderate comorbidities and a median age of 64 years old. The majority of them (75.3%) had FIGO stage IIIC disease, while one-fourth (24.7%) presented with peritoneal metastases (FIGO stage IV). Most patients had endometrioid, high-grade tumors, with substantial lymphovascular space invasion (LVSI) and deep myometrial invasion. Complete gross resection was achieved in 92.1% of the patients. NACT was administrated in 14.6% of the population. Deep myometrial invasion and non-endometrioid histology were recognized as independent prognostic factors for worse PFS, but no association was found for OS. Concerning survival rates, the median progression-free (PFS) and overall (OS) survival were 44 and 70 months, respectively. (4) Conclusions: Myometrial invasion and histological subtypes seem to affect the recurrence rate of advanced endometrial cancer patients. NACT could likely be an alternative for primarily inoperable and frail patients, but does not appear to alter survival rates.

## Simple Summary

This retrospective study evaluated prognostic factors and survival in 89 women with advanced endometrial cancer. The cohort consisted mainly of obese patients with moderate comorbidities and a median age of 64 years. Most patients had International Federation of Obstetrics and Gynecology (FIGO) stage III disease and one quarter had peritoneal metastasis (FIGO IV). Most tumors were high-grade endometrioid, with substantial lymphovascular space invasion and deep myometrial invasion. Complete gross resection was achieved in 92% of cases, while neoadjuvant chemotherapy (NACT) was administrated in 14.6% to facilitate cytoreduction. Median progression-free (PFS) and overall (OS) survival were 44 and 70 months, respectively. Non-endometrioid histology and deep myometrial invasion independently negatively affected PFS, whereas no clinicopathologic or treatment factor significantly affected OS. These findings suggest that tumor biology primarily drives recurrence risk in advanced endometrial cancer.

## 1. Introduction

Endometrial cancer (EC) is the most common gynecological cancer in high-income countries, usually affecting postmenopausal patients [1,2,3]. Its incidence is rising globally, even though mortality is steady [4,5]. In the United States, the estimated number of new cases and deaths were 66,200 (7%) and 13,030 (5%), respectively, in 2023 [1,6]. It is usually diagnosed early and confined to the uterus; however, approximately 21% of cases are presented at an advanced stage; 12% have regional or retroperitoneal lymph node spread and 9% of cases have distant metastases or peritoneal spread [7,8]. While survival rates for early-stage endometrial cancer are high, advanced endometrial cancer patients have a poorer prognosis [9,10]. Furthermore, in the era of molecular classification into five distinct categories (POLE-mutated; mismatch repair-deficient (MMRd); no specific molecular profile (NSMP) low-grade and estrogen-receptor-positive; NSMP high-grade or estrogen-receptor-negative (or both); and p53-abnormal (p53abn) [11,12]) advanced stage disease seems to impact patient prognosis irrespective of molecular status [13].

Surgery remains the cornerstone of endometrial cancer treatment. For early-stage disease total hysterectomy, bilateral salpingo-oophorectomy and surgical staging (possibly including infracolic omentectomy, lymphadenectomy, or sentinel lymph node biopsy) are the standard of care [14,15]. On the other hand, the treatment plan for advanced endometrial cancer (FIGO stages III–IV) is yet not fully established. Cytoreduction is proposed in order to achieve complete macroscopic resection. Resectability of the disease, at the time of diagnosis, plays a key role in the treatment algorithm and dictates the extent of surgery, similarly to ovarian cancer. Primary cytoreduction is the preferred approach for non-frail patients with resectable disease [11]. However, if the disease is deemed unresectable primarily, neoadjuvant chemotherapy (NACT) followed by cytoreduction could be an alternative option [11,16]. This treatment approach has been increasingly recommended for patients in recent years, but it has not been validated prospectively. All available data are from retrospective studies [17,18,19] and systematic reviews and meta-analysis [10,20,21].

Moreover, the prognostic factors impacting the survival rates of early-stage endometrial are well investigated, showing that age, high-grade tumors, deep myometrial invasion, substantial LVSI and p53-mutant expression independently impact survival rates [22,23,24]. However, in advanced endometrial cancer patients, data on predictors are scarce and vary across studies [18,19,25,26]. The aim of this study was to identify possible prognostic factors, especially the role of NACT, contributing to the survival rates for advanced endometrial cancer patients.

## 2. Materials and Methods

### 2.1. Study Characteristics

This retrospective analysis was conducted in the Gynecological Oncology Unit of the 1st Department of Obstetrics–Gynecology, AUTh, “Papageorgiou” General Hospital, from 1 January 2012 to 31 December 2023. We assessed the files of patients with endometrial cancer and identified those with advanced disease (FIGO stage III and IV) according to the updated 2023 FIGO staging classification. The conversion from the 2009 FIGO staging classification into the 2023 one was possible for all patients. When there was doubt about the final histology report, the slides were reviewed by a dedicated pathologist in gynecological oncology. Only patients with 2023 FIGO stage IIIC1ii and IIIC2ii were included in the final analysis. Women with presumed early-stage disease who were categorized into FIGO stage III due to microscopic lymph node metastasis during the staging procedure were excluded from the study. Initially, 308 patients were treated for endometrial cancer during this period of time. The study protocol was approved by the review board of our institution.

### 2.2. Patients

Inclusion criteria:

- Histological confirmation of advanced endometrial cancer.
- Surgical treatment at our Gynecological–Oncology Unit.

Exclusion criteria:

- Synchronous neoplasia at the time of diagnosis.
- FIGO stage IIIC1i and IIIC2i.
- Recurrent endometrial cancer.
- Missing important survival data.

As a result of the above-mentioned criteria, 205 out of the 308 women with endometrial cancer were excluded due to early-stage disease or pelvic and/or paraaortic microscopic lymph node metastasis. Moreover, 10 women were excluded because of recurrent disease or synchronous neoplasia and 4 were excluded due to missing important survival data. Finally, 89 women with advanced endometrial cancer were identified as eligible for further analysis.

### 2.3. Data Collection

Data were collected during a period of 30 days through the online registry of the Gynecological–Oncology Unit where all relevant data on patients’ medical records are stored. In order to avoid inconsistencies among different dates of data collection, a uniform data collection sheet (excel file) was used during the retrospective mining of the patient’s medical records. The data sheet included the following information:

- Patient hospital identification number;
- Patient age;
- Body Mass Index (BMI);
- Charlson Comorbidity Index (CCI);
- FIGO stage based on 2023 classification;
- Histological types;
- Tumor grade;
- Myometrial invasion;
- LVSI;
- Neo-therapies and adjuvant therapies;
- Residual disease after cytoreduction
- Time-related data:
  ○ Date of surgery;
  ○ Date of recurrence;
  ○ Date of the last follow-up or death.

### 2.4. Statistical Analysis

All statistical procedures were carried out using R (version 2025.09.0+387). Categorical variables were summarized as numbers with corresponding percentages, while continuous variables were described using the mean, median, interquartile range, and standard deviation. Progression-free survival (PFS) and overall survival (OS) were evaluated with Kaplan–Meier estimates, and both univariable and multivariable effects were examined with Cox proportional hazards models. PFS was defined as the time from surgery to the first evidence of recurrence or disease progression, and OS as the time from surgery until death or the last documented follow-up. A two-sided *p*-value less than 0.05 was considered indicative of statistical significance.

## 3. Results

Originally, 308 patients with endometrial cancer were identified and treated in the Gynecological–Oncology Unit, First Department of Obstetrics–Gynecology, Aristotle University of Thessaloniki, “Papageorgiou” General Hospital, from 2012 to 2023. After screening the patients based on the inclusion and exclusion criteria, 89 patients with advanced-stage disease were eligible for further analysis.

The patients’ characteristics are outlined in Table 1. The population of the study was obese, with moderate comorbidities and a median age of 64 years old. Most patients had FIGO stage III disease, while one-fourth suffered from peritoneal metastasis (FIGO stage IV). Moreover, the majority of the women (approximately 65%) had endometrioid, high-grade disease, while deep myometrial invasion of the uterus and lymphovascular space invasion (LVSI) was present in 66.3% and 40.4% of the population, respectively. Complete gross resection rates were high, up to 92.1%. The majority of the patients (45%) received concurrent or sequential chemoradiation. Neoadjuvant chemotherapy was administered to 13 patients (14.6%), of which 7 had peritoneal metastases (stage IV disease) and 6 had bulky lymph nodes (stage III disease). In addition, 74.2% of the patients received post-operative radiotherapy.

**Figure 1:**
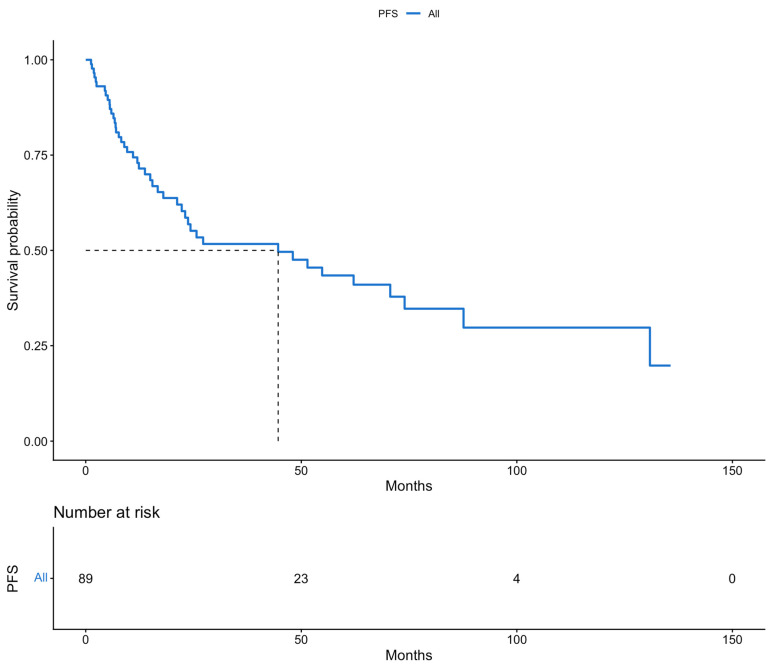
Progression-free survival (Kaplan–Meier curve).

**Figure 2:**
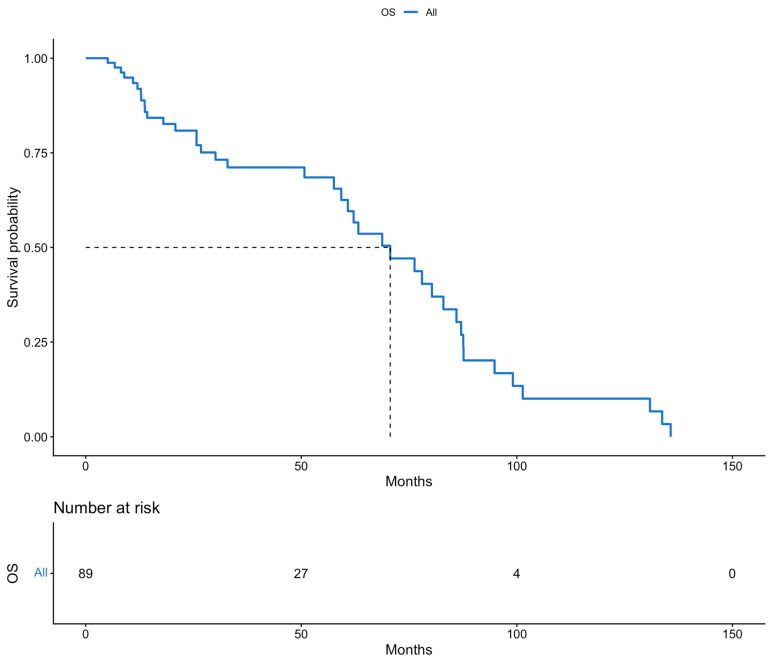
Overall survival (Kaplan–Meier curve).

The patients of this cohort were offered a close monitoring program, with a mean follow-up period of 36.2 months. Survival outcomes were estimated using Kaplan–Meier survival analysis. Firstly, the median survival rates were calculated. The median PFS of all patients included in the study was 44 months. Meanwhile, the median OS of the whole population of the study was 70 months. Secondly, we calculated the 5-year survival rates for both PFS and OS. The 5-year PFS and 5-year OS rates were 43.4% and 62.5%, respectively. These results are presented in Figure 1 and Figure 2.

The primary outcome of our study was to investigate possible prognostic factors affecting the survival rates of patients with advanced endometrial cancer. In order to identify these factors, a univariable analysis was conducted, including all the parameters that were collected for patients of this cohort. To avoid the effect of the confounders and to better select the independent factors affecting the survival rates of advanced-stage endometrial cancer patients, a multivariable analysis was performed.

Initially, Cox regression for the risk of recurrence was performed. In the univariable analysis, statistical significance was observed for age (HR: 1.04, 95% CI: 0.01–1.06, *p*-value < 0.05) and FIGO stage, specifically FIGO stage IV (HR: 2.85, 95% CI: 1.54–5.30, *p*-value < 0.05). Continuing with the multivariable analysis, factors with a *p*-value < 0.2 were included. Myometrial invasion and histology were identified as independent prognostic factors for PFS in advanced endometrial cancer patients. Specifically, deep myometrial invasion was associated with a 3.25-fold higher risk of recurrence or disease progression (HR: 3.25, 95% CI: 1.19–8.89, *p*-value = 0.0219) compared to myometrial invasion, which stood at <50%. Moreover, patients with non-endometrioid/other histology had a 2.54-folg higher risk of recurrence or disease progression (HR: 2.54, 95% CI: 1.18–5.44, *p*-value = 0.017) compared to those with endometroid histology. The aforementioned data are presented in detail in Table 2.

Furthermore, Cox regression for the risk of death was performed. In the univariable analysis, statistical significance was observed for adjuvant therapy, specifically in patients who were offered chemoradiotherapy (HR: 2.18, 95% CI: 0.17–10.50, *p*-value = 0.0363). Continuing with the multivariable analysis, factors with a *p*-value < 0.2 were included, but no independent prognostic factors were identified for OS in advanced endometrial cancer patients. The aforementioned data are presented in detail in Table 3.

**Figure 3:**
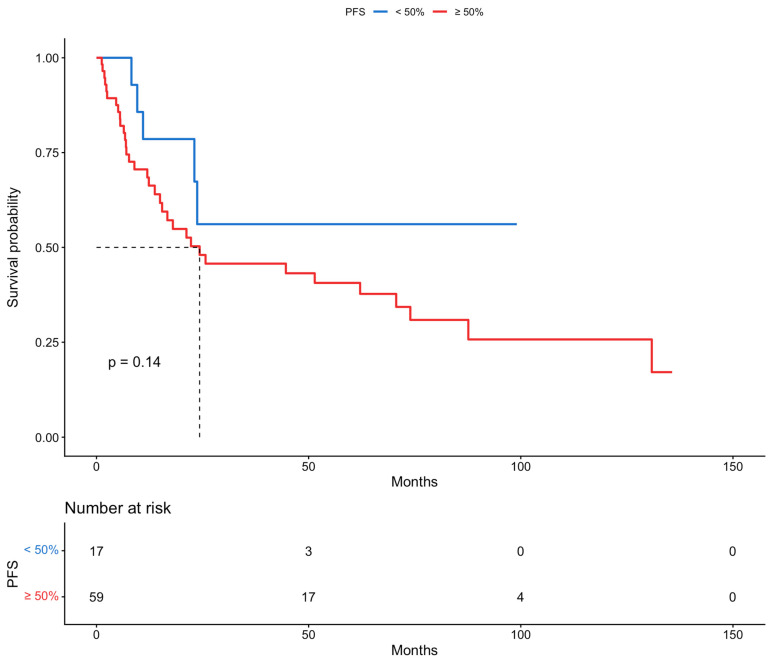
Progression-free survival, based on myometrial invasion (Kaplan–Meier curve).

**Figure 4:**
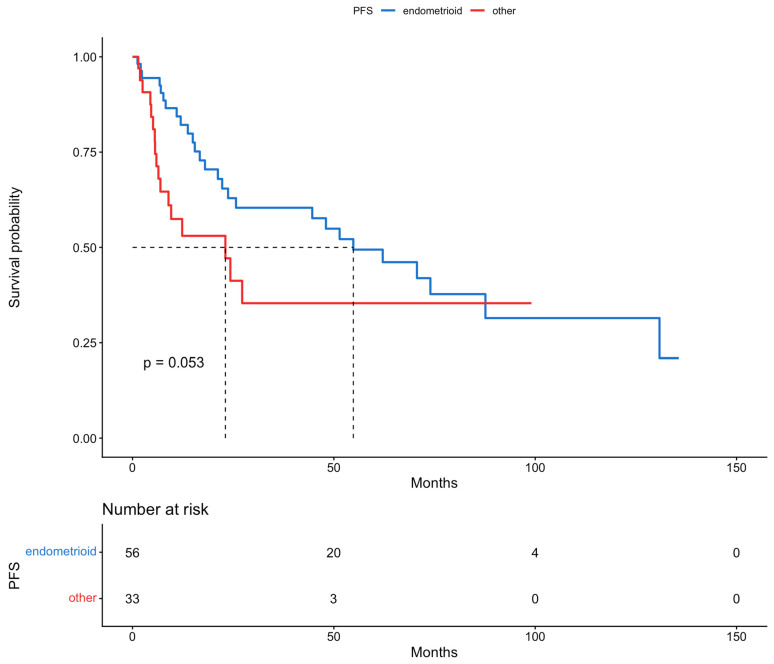
Progression-free survival, based on histology (Kaplan–Meier curve).

**Figure 5:**
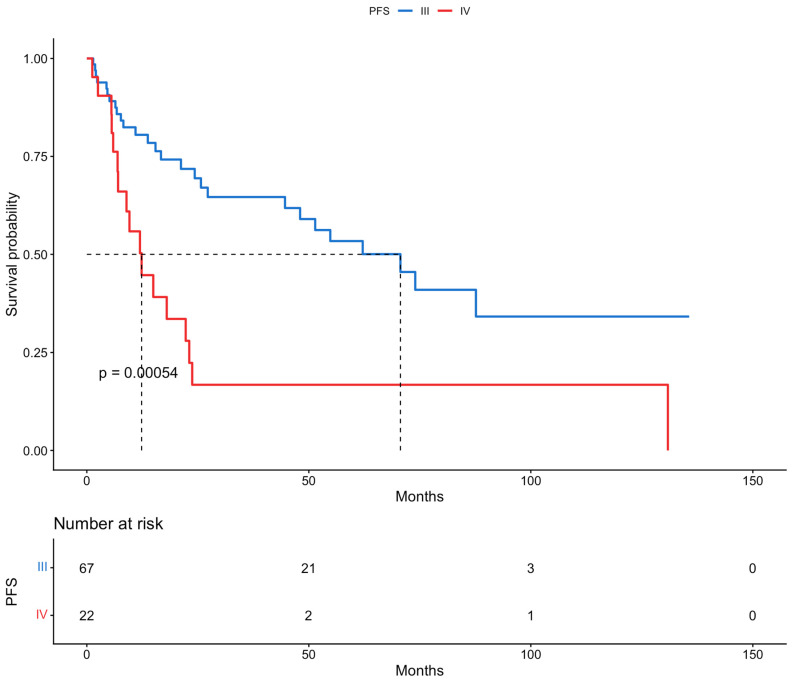
Progression-free survival, based on FIGO stage (Kaplan–Meier curve).

Based on the results of the multivariable analysis for the risk of recurrence or disease progression and the recognition of myometrial invasion and histology as independent prognostic factors for PFS, the population of this study was categorized according to myometrial invasion, information on which was available for 76 patients (<50%: 17 vs. ≥50%: 59), and histological subtypes (endometrioid, 56, vs. other, 33). Further survival analyses with Kaplan–Meier curves were performed. The median PFS rates in patients with <50% and ≥50% myometrial invasion were >136 and 24 months, respectively. Based on the histological subtypes the median PFS in patients with endometrioid and all other types of disease were 55 and 23 months, respectively. Differences in survival between the two groups were assessed by log-rank tests; statistical significance was not shown for both myometrial invasion (*p*-value = 0.14) and histology (*p*-value = 0.053). Moreover, PFS was assessed based on FIGO stage, and statistical significance was discovered (*p*-value < 0.05). The PFS for patients with FIGO stage III and stage IV was 71 and 12 months, respectively. These results are presented in Figure 3, Figure 4 and Figure 5.

## 4. Discussion

The primary objective of our study was to identify possible independent predictors affecting the survival rates of advanced endometrial cancer patients. Specifically, the role of NACT as a covariate in the univariable and multivariable survival analysis was investigated. This was based on the fact that while cytoreduction remains the cornerstone of these patients’ treatment, NACT is gradually implemented in the treatment algorithm.

We retrospectively analyzed 89 patients with advanced-stage endometrial cancer: 75.3% presented with FIGO stage III disease and 24.7% with FIGO stage IV disease. As it is believed that advanced endometrial cancer with peritoneal metastases and/or bulky lymph nodes should be treated similarly to ovarian cancer, the percentage of complete gross resection was as high as 92.1%, showing the importance of extensive debulking surgery. In total, 13 of the patients received NACT followed by cytoreduction; which were FIGO stage III and IV disease were almost equally distributed in these patients, but their overall percentage among the population of the study was relatively low (14.6%). Univariable and multivariable analysis revealed that myometrial invasion and histology were independent prognostic factors for PFS, but no factors were recognized for OS. Patients with deep myometrial invasion presented with worse PFS (24 vs. >136 months), while those with the endometrioid histological subtype showed an improved PFS (55 vs. 23 months), although a statistical significance was not reached, likely due to the limited sample size and follow-up. On the other hand, patients with FIGO stage IV disease, as expected, had significantly worse PFS (71 vs. 12 months). Interestingly, NACT was not a statistically significant covariate affecting either PFS or OS in the univariable and multivariable analyses. However, it is important to state that the patients who were deemed primarily inoperable, either due to high tumor load or frailty, formed a small percentage of the whole population of the study.

To our knowledge, only a few studies have investigated independent predictors in advance-stage endometrial cancer. Data on the possible prognostic factors contributing to the survival rates (PFS and OS) are scarce and sometimes contradictory [18,25,26]. Specifically, the role of NACT has been evaluated, through systematic reviews and meta-analyses [20,21,27] and a recent retrospective cohort study, only in the treatment of advanced endometrial cancer, but not as a predictor of survival, [19]. Moreover, in the recent update of the ESGO–ESTRO–ESP guidelines [11], for patients with advanced endometrial cancer with resectable FIGO stage III and IV disease, cytoreduction, including lymph node dissection of suspicious bulky nodes to achieve complete gross resection, is recommended. For frail patients or those with primary unresectable disease, especially MMRd tumors, irrespective of FIGO stage, NACT followed cytoreduction is proposed as an option, based on the experience gained from the treatment of advanced ovarian cancer.

A systematic review of nine studies [27] comparing primary cytoreduction and NACT included 5844 patients, of whom 22.5% received NACT. The data showed an increase in NACT administration from 2010 to 2015. Concerning overall survival, no difference was observed between the two groups, while pooled data analysis showed higher optimal cytoreduction rates for NACT followed by cytoreduction vs. primary cytoreduction (81.9% vs. 51.5%, respectively). These results differ significantly from those for our cohort, in which the percentage of NACT was much lower (22.5% vs. 14.6%) and the percentage of complete gross resection in both groups was >90%. This could be explained by the fact that our Gyneological Oncology Unit is ESGO-certified for advanced ovarian cancer surgery, ensuring high-quality complete cytoreductive surgery, because advanced endometrial cancer is surgically treated similarly to the way ovarian cancer is treated. On the contrary, a meta-analysis [20] of 16 studies including only patients with FIGO stage IV disease, with a total of 285 patients, found an NACT rate of 31%. Complete gross resection was achieved in 82% of patients, though primary cytoreduction led to better PFS and OS rates compared to NACT. These results are similar to our data, presenting high complete resection rates and no effect of NACT on the survival rates. Furthermore, two recent meta-analyses [10,21] investigating the survival benefit of complete cytoreduction in advanced endometrial cancer patients concluded that when complete gross resection is feasible, primary cytoreduction should be the only option, which is accordance with the results of our study. However, when there was residual disease after primary surgery, this was associated with poor survival rates. Albright et al. reported the existence of 18 studies with 1329 patients and a pooled complete gross resection rate of 52.1%, while the study by Pham et al. included 12 studies with 748 patients and found complete cytoreduction in 34.1%. Any residual disease was statistically significantly associated with worse PFS and OS in both studies. The most recent study investigating the role of NACT in advanced-stage endometrial cancer is a retrospective analysis of 51 patients integrating the new molecular classification [19]. NACT was administered in 29.5% of patients, with a complete gross resection rate of 40%, results that differ from those of our study. However, similarly to our conclusions, the administration of NACT was not recognized in that study as an independent prognostic factor of either PFS or OS.

Concerning the prognostic factors affecting the survival rates of patients with advanced-stage endometrial cancer, only a few studies in the literature provide clear information on this topic. A retrospective study [26] that included 69 patients with advanced-stage disease showed that only deep myometrial invasion was an independent prognostic factor for both PFS and OS, with no association with histology. The aforementioned data are in agreement with the results of our study concerning PFS. This could be attributed to the fact that deep myometrial invasion brings tumor cells into close proximity with the rich lymphovascular plexus in the outer myometrium and serosa, facilitating LVSI and subsequent nodal and distant dissemination in the peritoneal cavity [28,29]. The differences found in OS rates between our study and that by Mhawech-Fauceglia et al could be attributed to the main limitation of the latter study: the absence of patients with complete gross resection, which would have an important confounder in multivariable analysis. Conversely, a recent retrospective analysis of 80 advanced-stage endometrial cancer patients [18] with high (90%) complete gross resection rates showed that non-endometrioid histology was an independent prognostic factor for PFS and OS. This is partially in accordance with findings of our study, in which only PFS was statistically significantly affected in the multivariable analysis. The differences in OS between our study and that by Dinesh et al. could be attributed to the longer follow-up of patients in the latter study’s cohort compared to that in ours. In addition, the 10-year post hoc analysis of the PORTEC-3 trial, which included mostly FIGO stage III patients, showed that the molecular classification of endometrial cancer significantly affects PFS and OS, based on the adjuvant therapy. Last but not least, the main difference between our study and all three of the previous relevant publications is the absence of prognostic factors after the OS. This could possibly be explained by the small sample size, owing to the rarity of the disease itself, by the fact that OS data have not matured yet, due to the insufficiently long follow-up period, and by the fact that the majority of patients with intraperitoneal recurrences underwent surgery with high rates of zero residual disease.

The strengths of this study include the implementation of multivariate analyses for the recognition of possible prognostic factors affecting PFS and OS in advanced-stage endometrial cancer patients. Furthermore, our department is a university, and is tertiary ESGO-certified as an advanced ovarian cancer surgery center, ensuring a high level of quality indicators and surgical proficiency, which is also necessary for advanced endometrial cancer cytoreduction. This is also reinforced by the high (92.1%) complete gross resection rate of the population of our study. In contrast, the main limitations include its retrospective design and the lack of molecular classification. Another important confounder that could have affected the results of this study are the possible changes in the adjuvant treatment techniques and regiments of radiotherapy and chemotherapy used that could have occurred during the study period of 12 years. Additionally, external events such as the COVID-19 pandemic may have affected treatment pathways.

The principal contribution of this study to the literature is the addition of high-quality data from our ESGO-certified center for on rare disease. Our results are in agreement with previous published data from single centers and also add to the scarce amount of valuable data in the literature for possible future systematic reviews and meta-analyses. Future large prospective studies are needed in order to establish the aforementioned prognostic factors, while randomized control trials (RCTs) are needed for the clarification of the role of NACT in endometrial cancer, as with ovarian cancer.

## 5. Conclusions

Tumor biology seems to affect the recurrence rate of advance-stage endometrial cancer patients. Myometrial invasion and tumor histology were identified as independent prognostic factors for PFS, but not OS. Specifically, deep myometrial invasion (≥50%) significantly worsens PFS and the endometrioid histological subtype significantly improves PFS. Therefore, information on the presence and the depth of myometrial invasion is crucial for the patient’s prognosis and should always be reported in the final pathology report, even in advanced stages [30]. Furthermore, neoadjuvant chemotherapy did not appear to significantly alter survival rates as a predictor. NACT could possibly be an option for patients deemed inoperable primarily; however, future RCTs are needed to validate and provide robust data about the role of NACT in advanced-stage endometrial cancer.

## Figures and Tables

**Table 1:** Patient characteristics.

		Number of Patients (N)	Percentage (%)
Age (years)		median: 64	IQR: 57–78
BMI (kg/m^2^)		median: 29.9	IQR: 24.9–36.6
CCI		median: 3	IQR: 2–4
	0–2	31	34.9
	3–4	43	48.2
	≥5	15	16.9
Myometrial invasion			
	<50%	17	19.1
	≥50%	59	66.3
	unknown	13	14.6
LVSI			
	no	47	52.8
	focal	6	6.8
	substantial	36	40.4
Histological subtype			
	endometrioid	56	62.9
	other	33	37.1
Tumor grade			
	low	31	34.8
	high	58	65.2
FIGO stage			
	III	67	75.3
	IV	22	24.7
Adjuvant therapy			
	chemoradiation	40	45
	adjuvant chemotherapy	10	11.2
	NACT	13	14.6
	radiotherapy	26	29.2
Residual disease (cm)			
	0	82	92.1
	<1	2	2.3
	≥1	5	5.6

**Table 2:** Cox regression for recurrence.

		Univariate			Multivariate		
		HR	95% CI	*p*-Value	HR	95% CI	*p*-Value
Age (years)		1.04	1.01–1.06	**<0.05**	1.03	0.99–1.05	0.0658
BMI (kg/m^2^)		0.97	0.91–1.03	0.335			
CCI		1.06	0.93–1.21	0.377			
Myometrial invasion							
	<50%	1	1	1	1	1	1
	≥50%	2.00	0.78–5.15	0.148	3.25	1.19–8.89	**0.0219**
LVSI							
	no	1	1	1			
	focal	0.30	0.04–2.22	0.237			
	substantial	1.21	0.65–2.24	0.538			
Histological subtype							
	endometrioid	1	1	1	1	1	1
	other	1.84	0.98–3.42	0.0562	2.54	1.18–5.44	**0.017**
Tumor grade							
	low	1	1	1			
	high	1.58	0.81–3.06	0.18			
FIGO stage							
	III	1	1	1	1	1	1
	IV	2.85	1.54–5.30	**<0.05**	1.93	0.97–3.86	0.0625
Adjuvant therapy							
	chemoradiation	1.30	0.65–2.63	0.461			
	adjuvant chemotherapy	1.83	0.76–4.40	0.178			
	NACT	1.55	0.56–4.28	0.398			
	radiotherapy	1	1	1			
Residual disease (cm)							
	0	1	1	1			
	<1	1.19	0.21–2.19	0.815			
	≥1	0.67	0.27–5.23	0.507			

**Table 3:** Cox regression for death.

		Univariate			Multivariate		
		HR	95% CI	*p*-Value	HR	95% CI	*p*-Value
Age (years)		1.02	0.99–1.04	0.175	1.01	0.98–1.04	0.442
BMI (kg/m^2^)		0.97	0.92–1.06	0.693			
CCI		1.03	0.89–1.18	0.732			
Myometrial invasion							
	<50%	1	1	1			
	≥50%	1.10	0.48–2.52	0.819			
LVSI							
	no	1	1	1			
	focal	3.26 × 10^−8^	0.00–Inf	0.997			
	substantial	0.74	0.36–151	0.405			
Histological subtype							
	endometrioid	1	1	1			
	other	1.27	0.58–2.76	0.548			
Tumor grade							
	low	1	1	1			
	high	1.44	0.73–2.85	0.297			
FIGO stage							
	III	1	1	1			
	IV	0.85	0.37–1.93	0.693			
Adjuvant therapy							
	chemoradiation	2.18	0.17–10.50	**0.0363**	1.90	0.86–4.22	0.115
	adjuvant chemotherapy	0.97	0.33–2.88	0.9526	0.84	0.27–2.65	0.766
	NACT	0.75	0.10–5.87	0.7824	0.70	0.09–5.54	0.738
	radiotherapy	1	1	1	1	1	1
Residual disease (cm)							
	0	1	1	1			
	<1	0.74	0.17–3.27	0.693			
	≥1	0.85	0.32–2.26	0.75			

## Data Availability

In accordance with the journal’s guidelines, the data presented in this study are available on request from the corresponding author for the reproducibility of this study if requested.
